# Clinical utility of the INECO Frontal Screening for detecting Mild Cognitive Impairment in Parkinson’s disease

**DOI:** 10.1590/1980-57642018dn13-040005

**Published:** 2019

**Authors:** Yunier Broche-Pérez, Danay Bartuste-Marrer, Miriam Batule-Domínguez, Filiberto Toledano-Toledano

**Affiliations:** 1 MSc., PhD, Psychology Department, Universidad Central “Marta Abreu” de Las Villas, Santa Clara.; 2 Cuba and Cuban Initiative in Cognitive Health “CognitiON”.; 3 B.A, Arnaldo Milián Castro provincial Hospital, Santa Clara, Cuba.; 4 MD., MSc, Arnaldo Milián Castro provincial Hospital, Santa Clara, Cuba.; 5 Evidence-Based Medicine Research Unit, Hospital Infantil de México Federico Gómez National Institute of Health. Mexico City, Mexico.

**Keywords:** mild cognitive impairment, Parkinson’s disease, INECO Frontal Screening, cognitive screening., comprometimento cognitivo leve, doença de Parkinson, Rastreio Frontal INECO, triagem cognitiva.

## Abstract

Cognitive deficits in Parkinson’s disease typically affect executive functions. Recently, the concept of Mild Cognitive Impairment (MCI) has been related to PD (PD-MCI). PD-MCI is considered a transition phase to Parkinson’s disease Dementia. Therefore, it is important to identify PD-MCI in a reliable way. Objective: To evaluate the sensitivity and specificity of the INECO Frontal Screening (IFS) in detecting cognitive deficits in PD-MCI. Additionally, we compare the IFS and the Addenbrook Cognitive Examination Revised (ACE-R) between three groups; PD-MCI, MCI, and controls. Methods: The IFS and ACE-R were administered to 36 patients with PD-MCI, 31 with MCI (amnestic-multidomain subtype) and 92 healthy controls. Sensitivity and specificity were determined using ROC analysis. The groups were compared using one-way analysis of variance. Results: The IFS had adequate accuracy in differentiating patients with PD-MCI from healthy controls (AUC=0.77, sensitivity=0.82, specificity=0.77), and good accuracy in differentiating PD-MCI from MCI patients (AUC=0.80, sensitivity=0.82, specificity=0.61). However the IFS had low accuracy in differentiating MCI patients from healthy controls (AUC=0.47, sensitivity=0.52, specificity=0.41). On the ACE-R, the PD-MCI group had low performance in Fluency and Language. Only patients with PD-MCI had difficulties on the IFS, specifically in inhibitory control and visual working memory. This dysexecutive profile explains the sensitivity and specificity values found in the IFS. Conclusion: The present study results suggest that the IFS is a suitable screening tool for exploring cognitive dysfunction in PD-MCI, especially in those patients with a dysexecutive profile.

## RESUMO

Os déficits cognitivos na doença de Parkinson geralmente afetam as funções executivas. Recentemente, o conceito de Comprometimento Cognitivo Leve (CCL) tem sido relacionado à DP (DP-CCL). O DP-CCL é considerado uma fase de transição para a doença de Parkinson. Portanto, é importante identificar o DP-CCL de maneira acurada. Objetivo: Avaliar a sensibilidade e especificidade do *INECO Frontal Screening* (IFS) na detecção de déficits cognitivos na DP-CCL. Além disso, comparamos o IFS e o *Addenbrook Cognitive Examination Revised* (ACE-R) entre três grupos; DP-CCL, CCL e controles. Métodos: O IFS e o ACE-R foram administrados a 36 pacientes com DP-CCL, 31 com CCL (subtipo amnésico-de múltiplos domínios) e 92 controles saudáveis. A sensibilidade e especificidade foram determinadas usando a análise ROC. Os grupos foram comparados usando uma análise de variância unidirecional. Resultados: O IFS teve precisão adequada para diferenciar pacientes com DP-CCL de controles saudáveis ​​(AUC=0, 77, sensibilidade=0, 82, especificidade=0, 77) e boa precisão para diferenciar DP-CCL de CCL (AUC=0, 80, sensibilidade =0, 82, especificidade=0, 61). No entanto, o IFS teve baixa precisão para diferenciar CCL de controles saudáveis ​​(AUC=0, 47, sensibilidade=0, 52, especificidade=0, 41). No ACE-R, o grupo DP-CCL apresentou baixo desempenho em fluência e linguagem. Somente pacientes com DP-CCL apresentaram dificuldades no IFS, especificamente no controle inibitório e na memória de trabalho visual. Esse perfil disexecutivo explica os valores de sensibilidade e especificidade encontrados no IFS. Conclusão: O presente estudo sugeriu que o IFS é uma ferramenta de triagem adequada para explorar a disfunção cognitiva na DP-CCL, principalmente naqueles pacientes com perfil disexecutivo.

Mild cognitive impairment (MCI) identifies a transitional phase from cognitive changes of normal aging to those typically found in dementia, but with preserved activities of daily living.^1^ The prevalence of MCI ranges from 7 to 47.9%, with a worldwide average prevalence of 18.9 per 1000 population.^1^ Previous studies have shown that the annual conversion rate from MCI to Alzheimer disease (AD) was 10-15% and that approximately 50% of MCI patients will convert to AD within 4 years.^2^ Therefore, MCI is considered a transition phase from normal aging to AD.^2^

A few years ago, the concept of MCI was related to Parkinson’s disease (PD-MCI).^3^ Studies suggested that PD-MCI may represent the earliest stage of cognitive decline and may be a risk factor for developing Parkinson’s disease Dementia (PDD).^4^^,^ 5 Previous research estimated the prevalence of PD-MCI as lying in the range of 15% to 53% in PD.^6^ Diagnosing PD-MCI is an important issue because it predicts the development of dementia.^7^

The study of MCI and PD-MCI poses several challenges from a methodological point of view. Perhaps the most important is related to the presence of common risk factors for both pathologies. For example, age is one of the most important risk factors for the development of MCI, 1 whilePD is generally considered a disorder of older age, affecting between 1% and 2% of individuals older than 60 years.^8^

Usually, the investigations that are carried out in patients with PD-MCI presumethat the presence of cognitive decline is the result of the PD. However, we should bear in mind that the person exhibiting cognitive decline (possibly PD-MCI) would show signs of deterioration before receiving the diagnosis of PD, especially if the disease is diagnosed after the age of 60. The current results show that although the cognitive deficits present in AD and PD are heterogeneous, 9 ^,^ 10 there are cognitive impairment patterns that distinguish both pathologies. For example, cognitive deficits in PD typically affect executive functions, processing speed, attention and visuospatial abilities, 11 unlike AD, in which the main deficits are amnestic.^9^ In this sense, it is imperative to characterize PD-MCI not only in relation to PD without cognitive deterioration and PDD, but also to explore the cognitive characteristics that distinguish PD-MCI from MCI related to Alzheimer Disease.

There is also a need to validate the neuropsychological tests used to diagnose cognitive deficits associated with PD-MCI.^3^ Most studies published to date have used global batteries such as the Mini-Mental State Examination (MMSE), the Montreal Cognitive Assessment (MOCA), the Addenbrooke’s Cognitive Examination Battery (ACE), Mattis Dementia Rating Scale Second Edition (DRS-2) and Parkinson’s Disease-Cognitive Rating Scale (PD-CRS).^11^^,^ 12 These batteries are useful for obtaining a general picture of cognition in PD-MCI patients, but can also hinder the defining of deficits in specific cognitive dimensions (for example, executive dysfunctions).^12^^,^ 13

There is currently evidence that the INECO Frontal Screening (IFS) has good psychometric properties, including high internal consistency, for neurodegenerative pathologies, such as Alzheimer’s Disease (AD)^14^ and behavioral variant frontotemporal dementia (bv-FTD).^15^ In a sample of dementia patients and control subjects, Ihnen & Antivilo^16^ reported evidence of convergent validity showing significant correlations between the IFS and other instruments, such as the Frontal Assesment Battery, Wisconsin Card Sorting Test, and the Dysexecutive Questionnaire. For diagnostic accuracy, a cut-off point of 18 points (sensitivity=0.903; specificity=0.867) and an area under the curve of 0.951 was estimated for distinguishing between patients with dementia and control subjects. IFS has also shown utility in distinguishing patients with dysexecutive syndrome (bv-FTD) from patients with depression. A study conducted by Fiorentino, Gleichgerrcht^17^ revealed that the IFS had superior discriminatory accuracy (AUC=0.97) than both the MMSE (AUC=0.88) and ACE-R (AUC=0.93) in discriminating healthy controls from patient groups. 

The IFS is an easy-to-administer instrument for assessing several domains of executive function in a short time. The IFS comprises eight subtests arranged in three main domains of executive functions: 1) response inhibition and set-shifting, 2) working memory, and 3) capacity of abstraction.^16^ Additionally, performance on the IFS appears to be relatively independent of global cognitive functioning, suggesting specificity of IFS for executive functioning.^18^

The IFS had been used previously in PD patients with and without mild cognitive impairment (PD-MCI and PD-nMCI, respectively).^19^^,^ 20 In these studies, the sensitivity and specificity of the IFS in comparison with the MoCa was not determined. In this sense, it remains unclear whether the IFS is also a useful tool for exploring the features which distinguish PD-MCI from MCI related to Alzheimer Disease. 

In this study, we evaluate the sensitivity and specificity of the INECO Frontal Screening for detecting executive deficits in PD-MCI. Additionally, we compare the INECO Frontal Screening between three groups, PD-MCI, MCI, and controls.

## METHODS

### Participants

A total of 159 participants were evaluated in the period from January to September 2016. The sample was divided into 3 groups, comprising 31 patients with MCI, 36 patients with PD-MCI and 92 cognitively healthy controls. The participants included in the three groups were selected according to the criteria outlined below.

MCI-PD Group. Diagnosis of Idiopathic Parkinson’s disease, based on the UK PD Brain Bank criteria, 21 was established by a neurologist. Two clinical psychologists with neuropsychology training evaluated patients. Level I diagnosis of PD-MCI was established according to the MDS PD-MCI criteria^22^ if either the patient or an informant reported a cognitive decline, and using an abbreviated neuropsychological assessment. Patients were evaluated using the MMSE (patients scoring below 24 points were included). 

MCI Group (amnestic-multidomain subtype). We employed the criteria proposed by Petersen:^23^ significant impairment of the patient and/or caregiver’s report with objective evidence of memory decline compared to equivalent controls for age, sex, and years of education; Clinical Dementia Rating scale [CDR] score of 0.5, ACE-R scores<85 according to Cuban validation study, 24 and preservation of activities of daily living. Subjects with potential causes of cognitive decline other than neurodegenerative or cerebrovascular disease (e.g. depression, schizophrenia, epilepsy, head injury, alcoholism) were excluded. The ACE-R was used to determine MCI subtype, classifying the sample as amnestic-multidomain MCI subtype.

Control Group. The criteria for the control group were as follows: a score >85 on the ACE-R,^24^ no subjective memory complaints, preserved functioning in activities of daily living. Healthy controls had no neurological or psychiatric disorders.

None of the participants included in the study showed clinical signs of depression (Geriatric Depression Scale <5)^25^ or anxiety (Zung Anxiety Scale <51).^26^ Subjects with severe sensory deficits (vision or hearing) were also excluded.

### Instruments

The Addenbrooke Cognitive Examination Revised (ACE-R)^27^ consists of 5 components evaluating different cognitive domains, with separate scores: attention/orientation (18 points), memory (26 points), verbal fluency (14 points), language (26 points) and visuospatial functions (16 points), with a maximum score of 100 as the sum of scores of all domains. 

INECO Frontal Screening (IFS)^15^ is a neuropsychological exam to detect executive dysfunction in neurodegenerative pathologies. The tasks included in the IFS are *Luria motor series* (3 points), *Conﬂicting instructions* (3 points), *Go-no go* (3 points), *Months backwards* (2 points), *Backwards digit span* (6 points), *Modified Corsi tapping test* (4 points), *Proverb interpretation* (3 points) and *Modified Hayling Test* (6 points). The IFS has a maximum possible score of 30 points. High scores indicate preservation of executive functions.^28^

### Procedure and analysis of data

All participants received a full explanation of the research objective and subsequently signed the informed consent form. The cognitive evaluations were carried out by two clinical psychologists with neuropsychology training. The data were obtained observing the regulations of the Ethics Committee of the Universidad Central “Marta Abreu” de Las Villas, and in compliance with the Helsinki Declaration for Human Research.

The data were processed using SPSS/Windows, version 21. The three groups were compared using one-way analysis of variance and post-hoc comparisons using the Tukey HSD test. Effect sizes were calculated using partial eta squared (η^2^_partial_). Cohen classifies .01 as a small effect, .06 as a medium effect and .14 as a large effect.^29^ Linear regression was used to evaluate the effects of age and education on total scores of the ACE-R and INECO Frontal Screening. The values of sensitivity and specificity between groups were determined by the receiver operating characteristics (ROC) curve.

## RESULTS

### Demographics of PD-MCI, MCI and Control Groups

The present study included 36 patients with PD-MCI, 31 patients with MCI diagnosis and 92 cognitively healthy controls. The results of the comparison of demographics in the three groups are shown in Table 1. There were no significant differences in age or years of education among the three groups. Our results showed that, globally, age had no influence on the ACE-R (β=-0.024, *p=*0.77) or INECO Frontal Screening (β=-0.080, *p=*0.32), whereas years of education had a positive linear influence on the ACE-R (β=0.350, *p<*0.001) and INECO Frontal Screening (β=0.339, *p=*0.001). 

**Table 1:** Demographics of PD-MCI patients, MCI subjects and controls.

	MCI (n=31)	PD-MCI (n=36)	Control (n=92)	*p* (global)
Age (years)	74.2±8	72.6±8	73.1±7.1	.721
Education (years)	8.4±3.8	9.06±3.6	10±4.2	.344
Gender (male/female)^a^	20/11	21/15	62/30	-
Handedness (right/left)^a^	25/6	34/2	88/4	-

Values expressed as means±SD unless otherwise indicated;

a frequency.

### Validity of IFS for PD-MCI compared to ACE-R

**Figure 1:**
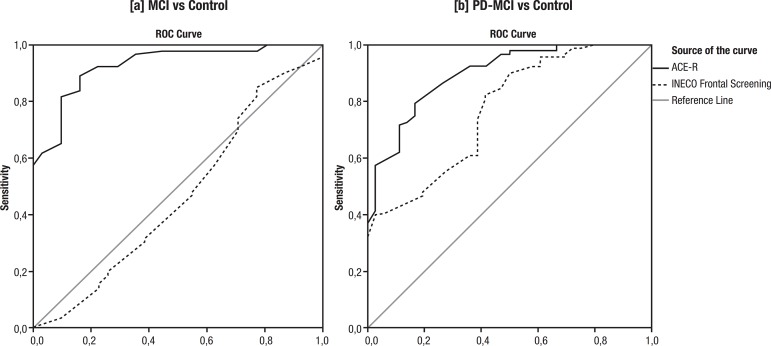
ROC curves of ACE-R (total score) and INECO Frontal Screening (total score) for detecting [a] MCI and [b] PD-MCI.

Figure 1 depicts ROC curves of the ACE-R (total score) and INECO Frontal Screening (total score) for detecting: a) MCI; and b) PD-MCI. When comparing MCI patients with the control group, the area under the ROC curve of the ACE-R was 0.92 (cutoff=84; sensitivity=0.90; specificity=0.76), while the area under the ROC curve of the IFS was 0.47 (cutoff=11; sensitivity=0.52; specificity=0.41) (Table 3). The same analysis was conducted between PD-MCI and controls. The results showed that the area under the ROC curve of the ACE-R for MCI-PD patients was 0.90 (cutoff=76; sensitivity=0.82; specificity=0.77), while the area under the ROC curve of the IFS was 0.77 (cutoff=21; sensitivity=0.90; specificity=0.72).

**Table 2:** Diagnostic accuracy of the ACE-R (total score) and INECO Frontal Screening (total scores and sub-scores) for the MCI group, PD-MCI group and between PD-MCI/MCI patients.

	MCI/Control				PD-MCI /Control				PD-MCI /MCI		
	AUC	CI (95%)	p		AUC	CI (95%)	p		AUC	CI (95%)	p
ACE-R (total)	0.92	0.89 ; 0.98	<.001		0.90	0.84 ; 0.95	<.001		0.49	0.34 ; 0.62	.82
INECO FS (total)	0.47	0.33 ; 0.58	.52		0.77	0.68 ; 0.85	<.001		0.80	0.68 ; 0.90	<.001
Go-No go	0.52	0.40 ; 0.64	.68		0.64	0.52 ; 0.78	.009		0.66	0.51 ; 0.80	.032
Modified Corsi Tapping	0.57	0.46 ; 0.70	.22		0.71	0.61 ; 0.81	<.001		0.77	0.64 ; 0.88	<.001
Modified Hayling Test	0.46	0.34 ; 0.60	.47		0.81	0.73 ; 0.90	<.001		0.80	0.63 ; 0.90	<.001

ACE-R: Addenbrook Cognitive Examination-Revised; INECO FS: INECO Frontal Screening; AUC: Area Under Curve; CI: Confidence Interval.

**Table 3:** Scores on the MMSE, ACE-R (total score and sub-scores), INECO Frontal Screening (total score and sub-scores) in PD-MCI, MCI, and control groups.

		MCI (n=31)	PD-MCI (n=36)	Control (n=92)	F	*p* (global)	h^2^_partial_
**ACE-R**	Attention and orientation	13.2±2	16±2.3	17.1±1.2	64.46	<.001	0.45
	Memory	12.4±3.2	17±6.1	18±4.1	14.7	<.001	0.20
	Fluency	7±2	4.4±2	9±2.4	53.2	<.001	0.40
	Language	21±4	13.4±4.4	24±3.3	103.2	<.001	0.56
	Visuospatial	11.2±2.2	11.1±4	14±2.1	17.3	<.001	0.18
	Total	63±10	61.3±14	81±9	64.6	<.001	0.45
**INECO Frontal Screening**	Luria motor series	2.6±.80	2.2±1.2	2.6±.80	2.18	.26	0.21
	Conﬂicting instructions	2.10±1.03	1.8±1.2	2.16±.94	0.84	.54	0.01
	Go-No go	1.8±1.16	1.08±1.4	1.7±1.02	4.82	.03	0.05
	Backwards digit span	2.4±2.09	2±1.4	2.41±1.7	1.02	.18	0.01
	Months backwards	1.68±.90	1.4±.90	1.53±.71	1.04	.41	0.01
	Modified Corsi tapping test	2.29±1.1	1.22±1.04	2.01±1.03	10.04	<.001	0.11
	Proverb interpretation	2.2±.90	1.8±1.2	2.4±0.9	2.79	.06	0.03
	Modified Hayling test	4.1±1.6	2±1.7	4.3±1.7	24.28	<.001	0.23
	Total	19.3±5.1	13±6.1	19±5	19.8	.016	0.20

Values expressed as means±SD unless otherwise indicated

**Figure 2:**
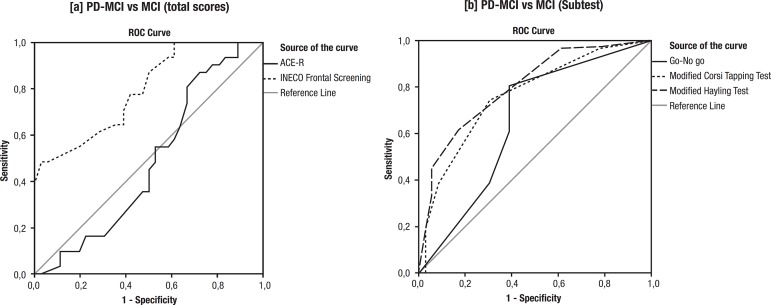
ROC curves show [a] ACE-R (total score) and INECO Frontal Screening (total score) for distinguishing between PD-MCI/MCI and [b] INECO Frontal Screening (sub-scores) for distinguishing between PD-MCI/MCI.

Figure 2 depicts ROC curves of: a) ACE-R (total score) and INECO Frontal Screening (total score) for distinguishing between PD-MCI/MCI; and b) INECO Frontal Screening (sub-scores) for distinguishing between PD-MCI/MCI. When comparing PD-MCI patients with the MCI group, the area under the ROC curve of the ACE-R was 0.49 (cutoff=43; sensitivity=0.52; specificity=0.68), while the area under the ROC curve of the IFS was 0.80 (cutoff=18, sensitivity=0.82, specificity=0.61) (Table 2). 

An additional analysis was conducted comparing the IFS subtest between the two groups. The results showed that the area under the ROC curve of the *Go-No go* subtest between PD-MCI/MCI patients was 0.66 (cutoff=0.5; sensitivity=0.85; specificity=0.38), while the area under the ROC curve of the *Modified Corsi Tapping* test was 0.77 (cutoff=0.5; sensitivity=0.97; specificity=0.77). Finally, the area under the ROC curve of the *Modified Hayling Test* was 0.80 (cutoff=0.5; sensitivity=0.94; specificity=0.70).

### Neuropsychological performance among PD-MCI, MCI and Control Groups

The results of the comparison of neuropsychological batteries among the three groups are shown in Table 3. Post-hoc comparisons using the Tukey HSD test indicated that mean MMSE scores differed significantly between both clinical groups (PD-MCI and MCI) in comparison to the healthy group (*p<*0.001). The MCI group had a significantly lower score on the MMSE compared to the PD-MCI group (*p<*0.001).

The means cores on the *Attention and Orientation* domain of the ACE-R showed significant differences in the MCI patient groups compared to the PD and Control groups (*p*<0.001). Patients with MCI performed worse than PD-MCI patients and healthy controls on the memory component of the ACE-R tests (*p*<0.001). *Fluency* scores also differed among the three groups. The mean score for the PD-MCI group differed significantly from the score in the MCI group (*p*<0.001). The control group also differed significantly from both MCI and PD-MCI groups (*p*<0.001).

The post-hoc comparisons also revealed differences among the three groups on the language domain. However, patients with PD-MCI had significantly lower scores on the language domain than MCI patients and cognitively healthy controls (*p*<0.001). Concerning the performance on the visuospatial component of the ACE-R, patients with PD-MCI and MCI performed worse than healthy controls (*p*<0.001), although there were no differences between these two groups (PD-MCI vs. MCI; *p*=0.98). Regarding differences in the *ACE-R total score*, both the PD-MCI and MCI groups performed worse than healthy controls (*p*<0.001), but no differences between them were found (PD-MCI vs. MCI; *p*=0.80).

No significant differences among groups were found on the INECO Frontal Screening for the following subtest: *Luria motor series*, *conflicting instructions*, *backward digit span*, *months backward* and *proverb interpretation* (Table 2). Post-hoc comparisons revealed significant differences on the *Go-No go task*, *Modified Corsi tapping* test, *Modified Hayling test*, and *IFS total score*. 

The PD-MCI patients had significantly lower scores on the *Go-No go task* relative to MCI patients (*p*=0.012) and cognitively healthy controls (*p*=0.03). No significant differences between the MCI and control groups were found (*p*=0.95). On the *Modified Corsi tapping* test, the PD-MCI group had significantly lower scores than the MCI group and controls (*p*<0.001). No differences were found between MCI patients and controls on this subtest (*p*=0.41). Significant differences were found on *the Modified Hayling test* and *Total IFS* between the PD-MCI group and the other two groups, while no significant differences between the MCI and control groups were found for these two variables.

## DISCUSSION

In this study, we evaluated the sensitivity and specificity of the INECO Frontal Screening in detecting cognitive deficits in PD-MCI. Additionally, we compared cognitive performance on the INECO Frontal Screening among the three groups: PD-MCI, MCI, and controls. 

Concerning the usefulness of the ACE-R and the INECO Frontal Screening, it was found that in MCI patients, the ACE-R possessed a high sensitivity and diagnostic specificity. These results confirm previous findings reporting the clinical utility of ACE-R in the diagnosis of MCI.^30^ However, the IFS showed low sensitivity and specificity for the MCI group.

Both the ACE-R and the IFS showed adequate diagnostic sensitivity and specificity for the detection of the cognitive deficits present in the PD-MCI patients in comparison with healthy controls. There is current evidence that Addenbrooke’s Cognitive Examination Revised (ACE-R) and the ACE-III have very good diagnostic sensitivity for cognitive decline in Parkinson disease.^31^^-^ 35 In the study conducted by Reyes et al., 31 a cut-off point of 83 points was reported (sensitivity=92%; specificity=91%;) to detect cognitive deficits in PD. Our results showed a similar cut-off point to distinguish PD-MCI from healthy controls, with high sensitivity (sensitivity=0.90), but lower specificity (specificity=0.76) compared to the study of Reyes et al.^31^ This may be because we used a sample of patients who met the specific clinical criteria for the diagnosis of PD-MCI, while the cited study used a sample of patients with PD, not specifying the type of cognitive deficit present in the sample. In another study conducted by McColgan et al.^34^ the sensitivity and specificity of the ACE-R for detecting PD-MCI were 69% and 84%, respectively, with a cut-off score of 89 points. Recently, Berankova et al.^35^ reported that the ACE-R had a cut-off score of 85.5 points (sensitivity: 68%, specificity: 91%) in discriminating PD-MCI from PD with normal cognition and 82.5 points (sensitivity: 70%, specificity: 73%) in discriminating PD-MCI from PDD.^35^

Compared to the ACE-R, the IFS showed better sensitivity and specificity when discriminating between the patients from the PD-MCI groups and the MCI patients. In particular, the IFS sub-tests that showed the best sensitivity and diagnostic specificity were those related to the processes of visual working memory and inhibitory control. This result may be related to the fact that cognitive deterioration in PD begins with impairments on tests that are sensitive to frontal lobe dysfunction, and then progresses with deficits on tests that involve more posterior cortical areas.^36^

In our results, the ACE-R showed low precision for discriminating between PD-MCI and MCI associated with Alzheimer’s (cut-off=43; sensitivity=0.52; specificity=0.56), while the area under the ROC curve of the IFS was 0.80 (cut-off=18, sensitivity=0.82, specificity=0.61). Other investigations have indicated that the IFS may be used as a screening test for executive dysfunction in other neurodegenerative conditions, such as Alzheimer Disease (AD)^14^^,^ 37 and behavioral variant Frontotemporal Dementia (bv-FTD).^17^

The tasks that showed the greatest ability to discriminate between PD-MCI and MCI patients are those that explore inhibitory control (Go-No Go and Modified Hayling test)and visual working memory (Modified Corsi tapping test). With regard to working memory, there are previous studies reporting that patients with mild-to-moderate PD were impaired on a test of visuospatial WM, while their performance on an analogous test of verbal WM was unaffected.^38^^,^ 39

In the case of the inhibitory control evaluated by the IFS using the Modified Hayling test, our results confirm the findings obtained by other studies that have explored patients with PDD. For example, a recent study investigated inhibitory control in people with Alzheimer’s disease dementia (ADD) and patients with Parkinson’s disease using the Hayling Sentence Completion Test (HSCT). According to some authors, the inhibitory control difficulties present in patients with Parkinson’s disease could be a characteristic with prognostic value to determine the risk of dementia.^40^

In relation to the second objective of the study, significant differences were found in MCI group compared with the other groups, in the Attention and Orientation domains, and Memory. The difficulties found in Language and the global score on the ACE-R did not differ from those attained by the PD-MCI group, but were both worse than the performance shown by the control group. The results obtained support the presence of a continuum of cognitive function within the concept of MCI, even when the main impairment affects memory.^2^

In PD-MCI patients, difficulties were detected in the fluency and language domains evaluated by the ACE-R, results that were significant in comparison with the other groups. These results support the findings of other studies reporting deficits in language among patients with PD. Previous research indicates difficulties in several processes related to language, such as processing action-related verbs, 41 sentences^42^ and deficits in the appraisal of action meanings evoked by naturalistic texts.^19^

Regarding the results achieved on the INECO Frontal Screening, difficulties were found in the executive functions by the PD-MCI groups that were significant in comparison with the other groups included in the study. The executive tasks on which the PD-MCI patients had difficulties were related to inhibitory control and visual working memory. Additionally, total score on the IFS was statistically lower in the PD-MCI group than the MCI or healthy groups. The MCI group presented no difficulties on any of the executive domains evaluated by the IFS, showing similar performance compared to healthy controls.

Our results are consistent with other studies assessing the most common MCI subtypes and their associations with later development of dementia in PD.^13^ While in MCI cases with memory deficits predominate, in PD-MCI most patients present the non-amnestic subtype exhibiting impairments in a range of cognitive domains, such as executive function, attention, processing speed, visuospatial ability, among others.^6^^,^ 13 ^,^ 19 ^,^ 43 ^,^ 44 This variety of affected functions could be related to the brain dysfunction patterns that have been proven in PD-MCI patients, characterized by hippocampus, prefrontal, occipital, and parietal brain atrophy.^45^^,^ 46 In our study, the cognitive profile that characterized the patients was dysexecutive. This dysexecutive profile found in PD-MCI patients is a factor that allows us to explain the values ​​of diagnostic sensitivity and specificity described for the IFS.

The present study had some limitations. First, our PD-MCI and MCI samples are relatively small, with only 36 PD-MCI cases, and 31 MCI patients. In future studies, large samples are needed to confirm statistically significant differences between diagnostic instruments. Second, we were unable to explore the relationship between neuropsychological assessments and other biomarkers in the MCI and PD-MCI patients because of economic and technological limitations. Consequently, the clinical diagnosis of MCI and PD-MCI were based on a comprehensive diagnostic procedure as the ultimate gold standard. 

In conclusion, this is the first study comparing performance on the IFS in subjects with MCI related to Alzheimer’s disease, PD-MCI patients, and healthy older controls. Our results showed that the IFS has a low capacity for discriminating between patients with MCI and healthy controls, while the ACE-R possesses a high diagnostic capacity to differentiate between these groups. In PD-MCI patients, both the IFS and the ACE-R have high sensitivity and diagnostic specificity when compared with healthy controls.

The ACE-R did not display discriminatory capacity to differentiate between PD-MCI patients and MCI related to Alzheimer’s disease. The IFS had adequate capacity to discriminate between PD-MCI and MCI patients related to Alzheimer’s disease, specifically the subtests of visual working memory and inhibitory control. These results suggest that the joint use of IFS and global screening tools for the cognitive investigation of PD-MCI would allow detection of the cognitive deficits present in these patients, facilitating early intervention and preventing MCI conversion to dementia. 
